# Seeking care where they can: A systematic review of global trends in online alcohol treatment utilization among non-veteran and veteran women

**DOI:** 10.1016/j.dadr.2022.100116

**Published:** 2022-11-17

**Authors:** Tracy Simpson, Rebecca Sistad, Jack T. Brooks, Noam G. Newberger, Nicholas A. Livingston

**Affiliations:** a Center of Excellence in Substance Addiction Treatment & Education, VA Puget Sound Healthcare System, Seattle, WA, USA; b University of Washington School of Medicine, Seattle, WA, USA; c VA Boston Healthcare System, Boston, MA, USA; d Boston University Chobanian & Avedisian School of Medicine, Boston, MA, USA; e National Center for PTSD, Behavioral Science Division, VA Boston Healthcare System, Boston, MA, USA

**Keywords:** Women, Online, Web-based, Alcohol, Treatment, Systematic review

## Abstract

**Background:**

Findings from a person-level meta-analysis of online alcohol intervention trials suggest that women disproportionately seek out such interventions (Riper et al., 2018). Although women may be a “hidden population” that is particularly drawn to online alcohol interventions, trial design features may explain women's apparent over-representation in these trials.

**Methods:**

This systematic review examined associations between gender-tailored recruitment/inclusion criteria and proportions of women enrolled in online alcohol intervention trials, evaluated whether community samples have greater proportions of women than clinical samples, and compared country-specific average proportions of women in trials to country-specific proportions of women with Alcohol Use Disorder (AUD).

**Results:**

Forty-four trials met inclusion/exclusion criteria, 34 community samples and 10 drawn from clinical settings; 4 studies included U.S. veterans and were examined separately. The average proportion of community-recruited women across the studies was 51.20% and the average proportion of clinically-recruited women was 35.81%, a difference that was statistically significant. Across the countries with relevant trials, the expected proportion of those with AUD who are women is 27.1% (World Population Review, 2022). Only 2 studies used targeted recruitment for women so no between-group tests were conducted. There was not a statistically significant difference in the proportion of women across trials that did and did not use gender-tailored alcohol inclusion criteria.

**Conclusions:**

Results from this systematic review suggest that study design factors do not account for the marked over-representation of women in online alcohol interventions, indicating that women are indeed a “hidden population” whose needs should be understood and accommodated.

## Highlights

• This review evaluated enrollment and trial design factors in online alcohol trials.
• Women have disproportionately high enrollment in online alcohol treatment trials.
• The expected proportion is 27%, but 51% of community-recruited samples are women.
• Gender-tailored recruitment and alcohol inclusion criteria do not account for this.

## 1 Introduction

Alcohol use and hazardous drinking remain critical public health concerns worldwide (World Health Organization [WHO], 2018). Men are generally understood to drink at higher levels than women and are also more likely to meet criteria for alcohol use disorder (AUD; Centers for Disease Control and Intervention, 2022; Grant et al., 2015). Analysis of WHO 2016 surveillance data support that prevalence of AUD among women were lower than men's in every one of the 188 countries that contributed data (World Population Review, 2022). The largest gender prevalence discrepancies were in Hungary and Russia (29.7% and 29.5%, respectively) and the smallest were in Qatar, Libya, and Saudi Arabia (0.3% for Qatar and 0.4% for the other two countries), with an average worldwide discrepancy of 6.7% (men's worldwide average: 8.8%; women's worldwide average: 2.1%). Even in the country with the highest prevalence of AUD among women, the United States (U.S.), there was an appreciable difference between men's (17.6%) and women's (10.4%) rates of AUD (difference = 7.2%). A large systematic review of 694 data sources found that globally, alcohol use was much more likely to be associated with age-standardized mortality among men than women (6.8% and 2.2%, respectively) with the same pattern apparent for disability adjusted life years among those aged 15 to 49 (men 8.9%; women 2.3%; GBD 2016 Alcohol Collaborators, 2018). Recent evidence, however, suggests that gender gaps may be diminishing globally (Slade et al., 2016), including in the U.S., particularly among younger people (Grucza et al., 2018; White, 2020).

Given the historical and current disparities and higher prevalence of alcohol use and AUD among men, it is striking that women appear to be over-represented in studies of online treatments for hazardous drinking and AUD. Specifically, across over a dozen randomized clinical trials (RCTs) of online alcohol interventions originating in eight different countries included in a person-level meta-analysis, 52% of the participants were women (Riper et al., 2018).^1^ Benchmarked against the aforementioned 2016 WHO data, this proportion of women is over 20% higher than would be expected in that the proportion of those with AUD who are women in these eight countries is 30.31% (World Population Review, 2022). Additionally, this level of engagement in online alcohol interventions by women stands in contrast with other findings showing that women with AUD are consistently *less* likely to initiate traditional in-person alcohol treatment than are men (Alvanzo et al., 2014; McCrady et al., 2020; Simpson et al., 2020), suggesting that women may be a “hidden population” that could benefit disproportionately from the availability of online treatment options (Postel et al., 2005).

To better understand women's high rates of engagement in RCTs evaluating online alcohol interventions, the present systematic review updated and extended the prior meta-analysis’ search and focused specifically on aspects of study design that may contribute to women being over-represented in such study samples (e.g., gender-tailored recruitment efforts, gender-tailored alcohol entry criteria). In other words, we sought to ascertain whether the higher-than-expected proportion of women in these trials is an artifact of study methods or is more likely driven by women's differential desire for and utilization of online alcohol treatments.

Two findings from the earlier person-level meta-analysis underscore the importance of better understanding women's engagement in online alcohol interventions. First, women on average presented to these trials with substantially more serious alcohol issues than did men. Specifically, using gender-calibrated criteria, Riper et al. (2018) found that 40.1% of women and 28.8% of men engaged in recent heavy drinking upon study entry, and average Alcohol Use Disorder Identification Test (AUDIT; Saunders et al., 1993) scores at baseline were 16.6 and 14.2 for women and men, respectively. AUDIT scores range from 0 to 40 with scores from 8 to 12 suggesting hazardous/harmful drinking while scores 13 and over indicate a moderate to severe AUD (Babor et al., 2001; Källmén et al., 2019). Second, in contrast with the extant traditional, in-person alcohol treatment literature that has consistently found no between-gender differences in alcohol outcomes (Holzhauer et al., 2020; Magill et al., 2019; McCrady et al., 2020; Newberry et al., 2019), Riper et al. (2018) found that women benefited significantly less from these online interventions compared to men. This finding was echoed in a secondary analysis of a national implementation study of an online intervention for U.S. combat veterans with symptoms of PTSD and signs of unhealthy drinking (Livingston et al., 2021). In addition to women veterans having greater representation (22.5% of sample) than expected relative to the proportion of women in the veteran population (Department of Veterans Affairs, 2017), veteran men in this study, on average, decreased their drinks per drinking day by 51.8% (7.5 to 3.6) from baseline to 3-month assessment, while veteran women decreased theirs by only 33.3% (4.8 to 3.2). Notably, women arrived at nearly the same number of drinks per drinking day as men, which is an especially concerning outcome given that this is the upper limit of daily drinking for women and should not be their average drinks per drinking day (United States Department of Health and Human Services, and United States Department of Agriculture, 2010).

During the COVID-19 pandemic (WHO, 2020), changes in substance use and disruption in treatment services were reported worldwide. Between one-fifth and one-third of adults reported higher levels of alcohol use in countries including Australia (Stanton et al., 2020), Belgium (Vanderbruggen et al., 2020), Canada (Canadian Centre on Substance Use and Addiction, 2020), China (Ren et al., 2020), France (Rolland et al., 2020), Germany (Koopmann et al., 2020), Poland (Bartoszek et al., 2020; Błaszczyk-Bębenek et al., 2020; Chodkiewicz et al., 2020), the United Kingdom (Jacob et al., 2021), and the U.S. (Barbosa et al., 2021; Pollard et al., 2020; Rodriguez et al., 2020). A recent systematic review and meta-analysis involving 128 studies from 58 countries documented changes in alcohol consumption from before to during COVID-19 and found that amount of alcohol consumed and frequency of consumption increased more among women than among men as well as that greater numbers of children in the home and greater depression, anxiety, and distress were all associated with larger increases in consumption (Acuff et al., 2022).

At the same time, restrictions to traditional, in-person treatment were implemented in response to the COVID-19 pandemic, which adversely affected access to substance use treatment across the globe (see Narasimha et al., 2022 for a review). Stay-at-home orders and fear of contracting COVID-19 also contributed to reduced help seeking behavior (Gibson et al., 2022; Jackson et al., 2021; Livingston et al., 2022). The unfortunate consequence of the COVID-19 pandemic on access to substance use treatment further highlights the importance of effective alternatives to in-person treatment (Murthy and Narasimha, 2021). Online interventions provide a viable avenue for disseminating evidence-based support to individuals with substance use disorders unable or unwilling to use in-person treatment (Enggasser et al., 2021).

The present systematic review replicated and extended the earlier search by Riper et al. (2018) with the following exceptions: only studies including both women and men and that evaluated online alcohol interventions were included. In light of the consistent finding that women are less likely to seek in-person alcohol treatment than are men (Magill et al., 2019; McCrady et al., 2020), we first evaluated whether studies that recruited community samples have significantly greater proportions of women participants than those that recruited in clinical settings. Next, to ascertain whether the over-representation of women in the studies included by Riper et al. (2018) is apparent in the larger universe of available studies, we compared the country-specific average proportions of women in community RCTs to the country-specific proportions of women with AUD using the 2016 WHO surveillance data (World Population Review, 2022).

Assuming that the pattern of women's over-representation in community online alcohol intervention RCTs was found to persist across the updated search, we anticipated testing two *a priori* hypotheses to address the question of whether the disproportionate involvement of women is an artifact of trial design. Specifically, we hypothesized that 1) studies that used recruitment strategies targeting women would have significantly greater proportions of women than those that did not, and 2) that studies that used gender-tailored alcohol study inclusion criteria would have significantly greater proportions of women than those that did not. Finally, we explored whether proportions of women in these RCTs varied over time and tallied whether participant characteristics and outcomes were reported by gender. When gender specific participant characteristics and outcomes were reported, those findings are summarized. Studies that recruited U.S. veterans were considered separately in all inquiries because the proportion of women in these studies is constrained by the fact that women comprise a small proportion of the U.S. veteran population (i.e., approximately 9.4%; Department of Veterans Affairs, 2017).

## 2 Methods

### 2.1 Protocol and registration

No published protocol exists for this systematic review.

### 2.2 Eligibility criteria

The inclusion criteria for this systematic review were largely consistent with Riper et al. (2018) except that we required studies to not have excluded women or men. Additionally, studies needed to (1) be RCTs that compared an online intervention, targeting alcohol use, with a control or comparison condition;^2^ (2) the experimental intervention needed to be based on behaviorally oriented therapeutic principles, including personalized normative feedback, behavioral self-control, cognitive-behavioral therapy, and/or motivational interviewing; (3) study participants needed to be at least 18 years of age, and (4) they needed to have quantifiable levels of alcohol consumption that exceeded recommendations for low-risk drinking (we also included studies that used AUD diagnostic status or concern about drinking as their entry criterion). Like Riper et al. (2018), we excluded studies that targeted recruitment to university students or pregnant people and we allowed studies that used either an unguided or guided intervention or both. We excluded meta-analyses, reviews, commentary papers, and protocols. We also excluded secondary results papers of eligible studies.^3^ Articles were not eligible if they were not available in English or the full text was not available online.

### 2.3 Information sources

We followed the Preferred Reporting Items for Systematic Reviews and Meta- Analyses (PRISMA) standards for this study (Moher et al., 2009). The third author (JT) systematically searched the seven databases used by Riper et al. (2018): Pubmed, Embase, CINAHL, PsychInfo, Science Citation Index Expanded, Social Sciences Citation Index, Arts and Humanities Citation Index. Databases were searched from inception to November 9, 2021.

### 2.4 Search, study selection, and data collection process

Following Riper et al. (2018) example, we used the same controlled vocabulary terms and keywords appropriate to search each database for online alcohol intervention trials that met our inclusion/exclusion criteria (see Table 1 for full search terms used in each database). Once duplicates were removed, all articles were marked as either “potentially eligible” or “ineligible” by two authors, serving as independent raters, and all discrepancies were discussed and resolved by consensus of the first and last authors (TS and NL). The remaining studies underwent full text review by either the first or the last author (TS, NL) and by one of the other authors (JT, RS, NN). All discrepancies between screeners were discussed and resolved by consensus of the two screeners. Disagreements were settled through discussion until consensus was reached.

**Table 1:** Databases and search terms used

Database	Search Terms
Pubmed	("Internet"[All Fields] OR "Web"[All Fields] OR "online"[All Fields] OR "computer"[All Fields] OR "mobile"[All Fields] OR "internet"[MeSH Terms]) AND ("self-help"[All Fields] OR "brief intervention"[All Fields] OR "treatment"[All Fields] OR "unguided"[All Fields] OR "guided"[All Fields] OR "supported"[All Fields] OR "low-intensity"[All Fields] OR "Randomized Controlled Trials as Topic"[MeSH Terms] OR "treatment outcome"[MeSH Terms]) AND ("alcohol abuse"[All Fields] OR "dependence"[All Fields] OR "problem drinking"[All Fields] OR "hazardous drinking"[All Fields] OR "harmful drinking"[All Fields] OR "abstinence"[All Fields] OR "alcohol drinking"[MeSH Terms] OR "Alcoholism"[MeSH Terms]) AND ((randomizedcontrolledtrial[Filter]) AND (humans[Filter]) AND (english[Filter]) AND (alladult[Filter])) AND ((randomizedcontrolledtrial[Filter]) AND (humans[Filter]) AND (2017/5/31:2021/11/9[pdat]) AND (english[Filter]) AND (alladult[Filter]))
Embase	('internet' OR 'web' OR 'online' OR 'computer' OR 'mobile') AND ('self-help' OR 'brief intervention' OR 'treatment' OR 'unguided' OR 'guided' OR 'supported' OR 'low-intensity' OR 'randomized controlled trials as topic' OR 'treatment outcome') AND ('alcohol abuse' OR 'dependence' OR 'problem drinking' OR 'hazardous drinking' OR 'harmful drinking' OR 'abstinence' OR 'alcohol drinking' OR 'alcoholism') AND english:la AND ('randomized controlled trial' OR 'rct') AND adult AND [2017–2021]/py
CINAHL	('internet' OR 'web' OR 'online' OR 'computer' OR 'mobile') AND ('self-help' OR 'brief intervention' OR 'treatment' OR 'unguided' OR 'guided' OR 'supported' OR 'low-intensity' OR 'randomized controlled trials as topic' OR 'treatment outcome') AND ('alcohol abuse' OR 'dependence' OR 'problem drinking' OR 'hazardous drinking' OR 'harmful drinking' OR 'abstinence' OR 'alcohol drinking' OR 'alcoholism') AND AB 'alcohol' English Language; Randomized Controlled Trials; Age Groups: All Adult
PsychInfo	(Internet OR Web OR online OR computer OR mobile OR MA internet) AND (self-help OR brief intervention OR treatment OR unguided OR guided OR supported OR low-intensity OR MA Randomized Controlled Trials as Topic OR MA treatment outcome) AND (alcohol abuse OR dependence OR problem drinking OR hazardous drinking OR harmful drinking OR abstinence OR MA alcohol drinking OR MA Alcoholism)
Science Citation Index Expanded	'internet' OR 'web' OR 'online' OR 'computer' OR 'mobile' (All Fields) and 'self-help' OR 'brief intervention' OR 'treatment' OR 'unguided' OR 'guided' OR 'supported' OR 'low-intensity' OR 'randomized controlled trials as topic' OR 'treatment outcome' (All Fields) and 'alcohol abuse' OR 'dependence' OR 'problem drinking' OR 'hazardous drinking' OR 'harmful drinking' OR 'abstinence' OR 'alcohol drinking' OR 'alcoholism' (All Fields) and alcohol (Topic) and adult OR age OR years (All Fields) and English (Languages) and Articles (Document Types)

Screening and extraction were conducted using Covidence systematic review software, published by Veritas Health Innovation in Melbourne, Australia. Covidence automatically detected discrepancies in the extraction coding, which were primarily non-substantive (e.g., one coder capitalized intervention names and the other did not, differences in how many decimals were recorded, etc.). One of the two raters reviewed the discrepancies and either chose an entry for the final dataset or went back to the source document to adjudicate substantive discrepancies.

### 2.5 Data items

When available, the following data items were extracted from the articles that met our inclusion/exclusion criteria: overall sample size, number/percent of women, type of sample (community, clinical, veteran), alcohol entry criteria (including whether they were gender-tailored), whether there was mention of recruitment efforts targeting women, country or countries in which the study took place, year (or years) over which data collection took place, whether participant characteristics and/or outcomes were reported separately for women and for men, type of treatment, and number of modules or sessions delivered over time. For those studies that did report participant characteristics and/or outcomes by gender, those findings were recorded.

Additionally, in order to benchmark (contextualize) the proportions of women participants in the online alcohol RCTs with the expected proportions of women with alcohol concerns in their respective countries, we used country-specific rates of AUD from the WHO.^4^ To calculate the expected proportion of women with AUD for each country represented among the final set of studies, we added the estimated prevalence of women with AUD to the prevalence of men with AUD. We then divided the prevalence of women with AUD by the above sum as follows (using U.S. information as the example):% women (**10.4%**) +% men (**17.6%**) = **28.0%** 10.4% / 28.0% = **37.14%** To provide this same type of context for studies involving U.S. veterans, we used the above-derived expected proportion of women with AUD in the U.S. and multiplied this by the proportion of U.S. veterans who were women in 2015,^5^ which was 9.4% (Department of Veterans Affairs, 2017). This resulted in an expected proportion of 3.49% of the U.S. veteran population who are both women and had an AUD. Prior research on U.S. women veterans’ rates of alcohol problems, to include AUD, has consistently shown that they are on par with those of U.S. women in the general community (Hoggatt et al., 2015, 2017).

## 3 Results

### 3.1 Search and preliminary results

**Fig. 1:**
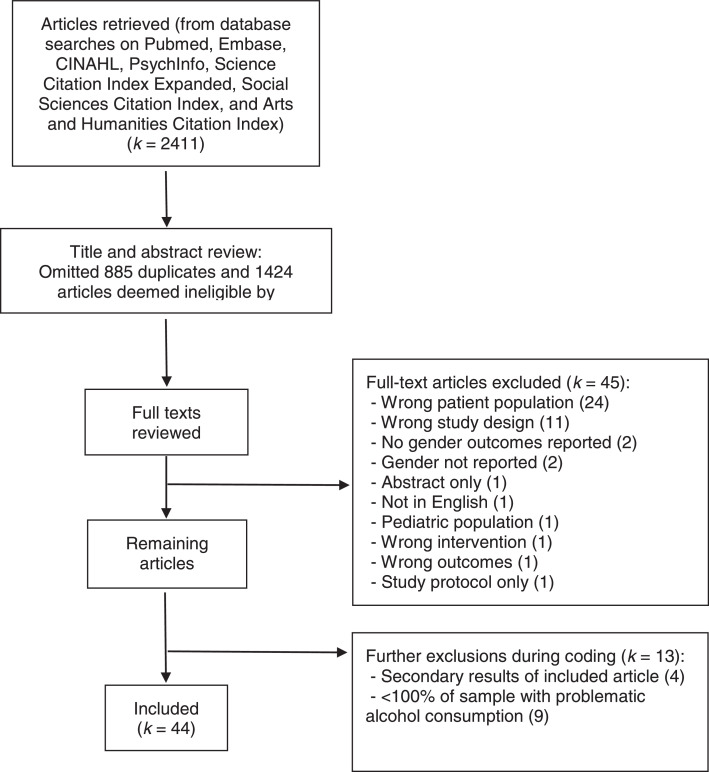
PRISMA diagram.

The search yielded 2411 articles (see Fig. 1). After the 885 duplicates were removed, 102 of the remaining 1424 articles met inclusion criteria for full text review. Upon closer review an additional 45 articles were excluded for not meeting inclusion/exclusion criteria (e.g., wrong study design, wrong patient population, etc.), leaving 57 articles for extraction. During coding, we omitted an additional 13 articles upon discovering the following issues: 1) reports of secondary results that were duplicative with primary outcome papers, 2) study samples not comprised solely of people reporting problematic alcohol use, or 3) intervention delivery modes that were not online or web-based. The remaining 44 articles described various online interventions for problematic alcohol use and included both women and men who screened positive for unsafe alcohol use (see Table 2). Thirty-four study samples were comprised of people recruited from communities or large work settings via broad advertising and 10 were comprised of people recruited from specific clinical settings, including substance use treatment programs, outpatient primary care and general medical clinics, and inpatient medical/surgical settings.

**Table 2:** Characteristics of Studies Included in Systematic Review.

Author	Publication Date	Country	Year(s) of Data Collection	Type of Sample	Alcohol Entry Criteria	% Women	Type of Intervention (experimental)	# of Modules/ Duration
Augsburger	2022	Estonia	2019	Community	AUDIT ≥ 8	48.90	CBT, MI	10/8 weeks
Baumann et al.	2017a	Germany	2008–2009	Community (job-seekers)	AUDIT-C ≥ 4/5 for F/M	36.20	TTM stage-tailored PNF vs. PNF	2/baseline & 12 weeks
Baumann et al.	2017b	Germany	2011–2012	Clinical (medical inpatient)	AUDIT-C ≥ 4/5 for F/M	25.18	PNF	2/baseline & 12 weeks
Baumgartner	2021	Switzerland, Germany, Austria	2016–2020	Community	AUDIT ≥ 8	48.33	CBT/MI for alcohol and depression vs. CBT/MI for alcohol only	8/6 weeks
Blankers et al.	2011	Netherlands	2008–2009	Clinical (SUD)	AUDIT ≥ 8 and ≥ 14 drinks/week	50.73	CBT/MI self-help vs. CBT/MI internet therapy	4/4 weeks vs. 7 sessions
Boß et al.	2018	Germany	2014–2016	Community (workers)	AUDIT ≥ 6/8 and ≥ 14/21 drinks/week for F/M	58.99	unguided MI/PNF vs. MI/PNF with adherence-focused guidance	5/5 weeks
Brendryen et al.	2017	Norway	2011	Community (workplace)	FAST ≥ 3	51.76	Self-regulation	62/6 months
Author	Publication Date	Country	Year(s) of Data Collection	Type of Sample	Alcohol Entry Criteria	% Women	Type of Intervention (experimental)	# of Modules/ Duration
Brendryen et al.	2014	Norway	2011–2012	Community	FAST ≥ 3	33.20	Self-regulation	62/6 months
Brief	2018	United States	–	Community (veterans)	AUDIT 5–25/8–25 for F/M and QDS < 3/4 drinks/occasion, 7/14 drinks/week for F/M	13.77	CBT, MI, self-control	8/8 weeks
Caballeria et al.	2021	Spain	–	Community (primary care)	AUDIT-C ≥ 4/5 for F/M	35.63	CBT, MI, self-control	3/freely accessible
Cougle	2017	United States	–	Community and Clinical (general medical)	≥ 4 DSM-5 AUD symptoms	68.97	Interpretation bias modification	8/4 weeks
Cunningham	2009	Canada	–	Community	AUDIT-C ≥ 4	47.03	PNF	1/1 sitting
Cunningham et al.	2017	Canada	–	Community	AUDIT ≥ 8	49.59	CBT, MI	20/freely accessible
Fernandez et al.	2019	United States	2012–2014	Emergency room	AUDIT-C ≥ 3/4 for F/M	60.40	Computer-delivered MI vs. therapist-delivered MI with computer guidance	8/1 sitting
Freyer-Adam	2018	Germany	2011–2012	Clinical (inpatient)	AUDIT-C ≥ 4/5 for F/M	24.72	In-person MI vs. computer-generated PNF letters	3/baseline, 4 & 12 weeks
Author	Publication Date	Country	Year(s) of Data Collection	Type of Sample	Alcohol Entry Criteria	% Women	Type of Intervention (experimental)	# of Modules/ Duration
Guillemont et al.	2017	France	2009	Community	AUDIT ≥ 6/7 for F/M	31.56	Behavioral counseling intervention	3/6 weeks
Hansen	2012	Denmark	2008	Community	≥ 14/21 drinks/week for F/M	44.86	Brief PNF vs. personalized brief advice	1/1 sitting
Haskins et al.	2017	United States	–	Emergency department	AUDIT ^a^	36.79	SBIRT	1/1 sitting
Hester et al.	2005	United States	–	Community	AUDIT ≥ 8	47.54	MI	3/1 sitting
Jo et al.	2019	South Korea	2017	Community	AUDIT-C ≥ 3/4 for F/M	48.13	PNF, MI	2/4 weeks
Johansson et al.	2021	Sweden	–	Clinical (AUD)	AUDIT ≥ 15	38.21	MI, behavioral self‐control	8/10–12 weeks
Johnson et al.	2018	Australia	2012	Clinical (general medical)	AUDIT-C 5–9	25.09	SBI	1/1 sitting
Jones et al.	2018	United Kingdom	–	Community	≥ 14/21 units for F/M	47.15	Inhibitory control training	14/4 weeks
Khadjesari	2014	United Kingdom	2012	Employees	AUDIT-C ≥ 5	24.51	SBI	1/1 sitting
Kiluk et al.	2018	United States	2012–2014	Clinical (AUD)	Past 30 days DSM-IV criteria for alcohol abuse or dependence	35.29	CBT vs. CBT + weekly clinical monitoring	7/8 weeks
Miller	2018	United States	–	Community (veterans)	AUDIT ≥ 3/4 for F/M	17.34	PNF	1/1 sitting
Mujcic	2020	United Kingdom	2007–2009	Community	AUDIT-C ≥ 5	61.23	CBT, MI	8/1 hour, 5 days, or 4 weeks
Author	Publication Date	Country	Year(s) of Data Collection	Type of Sample	Alcohol Entry Criteria	% Women	Type of Intervention (experimental)	# of Modules/ Duration
Pedersen	2017	United States	–	Community (veterans)	AUDIT ≥ 3/4 for F/M	16.71	PNF	1/1 sitting
Postel	2010	Netherlands	2008	Community	15–67/22–99 units/week for F/M	53.85	CBT	9/12 weeks
Riper	2008	Netherlands	–	Community	Past 3 months ≥ 14/21 per week or ≥ 4/6 units 1+ day/week for F/M	49.04	CBT, self-control	4/6 weeks
Sanatkar	2021	Australia	2014–2015	Community (young adults)	Past month ≥ 5 standard drinks in one sitting at least 2 times	61.31	CBT/MI vs. CBT/MI + clinician-guided digital forum	4/4 weeks, sessions available to review for 12 months
Schaub	2021	Brazil, Mexico, India, Belarus	2016–2019	Community	AUDIT ≥ 8	29.86	CBT, MI, self-control	8/6 weeks
Schulz et al.	2013	Germany	2010–2011	Community	≥ 1/2 drinks/day for F/M, drinking ≥ 5 days/week; AUDIT ≥ 7, or alcohol use while trying to get pregnant or pregnant/ breastfeeding, or trying to impregnate one's partner (men)	43.53	PNF	3/6 months
Simon	2019	United States	2016–2018	Clinical (primary care veterans)	AUDIT-C ≥ 3/4 for F/M and ≥ 3/4 drinks per day, 7/14 per week for F/M	11.24	MI	2/12 weeks
								
Author	Publication Date	Country	Year(s) of Data Collection	Type of Sample	Alcohol Entry Criteria	% Women	Type of Intervention (experimental)	# of Modules/ Duration
Sinadinovic et al.	2014	Sweden	2009–2010	Community	AUDIT ≥ 6/8 for F/M	54.98	PNF vs. CBT	1 vs. 18/freely accessible
Stapinski	2021	Australia	2017–2018	Community	AUDIT ≥ 8	67.48	CBT	5/8 weeks
Sundström	2020a	Sweden	2016–2017	Community	Past week ≥ 11/14 drinks for F/M, and ≥ 2 current DSM-5 AUD symptoms	51.20	CBT high vs. low intensity	13 v. 9/12 weeks
Sundström	2020b	Canada	2018	Community	AUDIT ≥ 8 and ≥ 14 drinks/week	64.71	CBT	20/12 weeks
Sundström et al.	2016	Sweden	2012–2013	Community	AUDIT ≥ 6/8 for F/M	60.00	Synchronous CBT vs. asynchronous CBT vs. unguided CBT	8/9 weeks
Tait	2019	Australia	2018	Community	AUDIT ≥ 7	70.74	CBT	4/12 weeks
Wallace	2011	United Kingdom	2007–2009	Community	AUDIT-C ≥ 5	57.00	CBT, MI	8/1 hour, 5 days, or 4 weeks
Wilks et al.	2018	United States	2016	Community	Past month ≥ 2 episodes of heavy episodic drinking (≥ 4/5 drinks for F/M in 2 h)	69.49	DBT	8/8 weeks
Wittleder et al.	2019	United States	–	Community (MTurk)	Self-concern about alcohol	53.50	Self-regulation	1/1 sitting
Zill	2019	Germany	–	Community	Avg. consumption of >12/24 g pure alcohol/day for W/M and /or AUDIT-C 3+	52.47	CBT	4/6 months

*Note*. Author names refer to the lead author's last name. AUD = Alcohol Use Disorder; AUDIT = Alcohol Use Disorders Identification Test; AUDIT-C = Alcohol Use Disorders Identification Test –Consumption; DAST = Drug Abuse Screening Test; DSM-5 = *Diagnostic and Statistical Manual of Mental Disorders* (Fifth Edition); DSM-IV-TR = *Diagnostic and Statistical Manual of Mental Disorders* (Fourth Edition, Text Revision); DSM-IV = *Diagnostic and Statistical Manual of Mental Disorders* (Fourth Edition); FAST = Fast Alcohol Screening Test; F = Female; M = Male; QDS = Quick Drink Screen; CBT = Cognitive Behavioral Therapy; MI = Motivational Interviewing; PNF = Personalized Normative Feedback; SBIRT = Screening, Brief Intervention and Referral to Treatment; SBI = Screening and Brief Intervention; DBT = Dialectical Behavior Therapy; TTM = Transtheoretical model of change.

^a^Specific cut-scores for the AUDIT were not provided: “Risky alcohol users were defined as having used alcohol above the AUDIT quantity or frequency guidelines.”.

Type, intensity, and duration of the online interventions was quite varied. Twenty studies included interventions based on principles specific to cognitive behavioral therapy, 17 interventions drew content from motivational interviewing, 11 delivered personalized normative feedback, 9 provided self-control or self-regulation exercises, and 3 offered screening and brief intervention. Fourteen interventions consisted of 1–3 modules generally delivered in 1 sitting, 12 interventions were delivered over 4–6 weeks, 11 over 8–12 weeks, 4 over 6 months, and 3 did not have a time limit.

Four studies focused exclusively on U.S. veterans – 3 community recruited studies (Brief et al., 2018; Miller et al., 2018; Pedersen et al., 2017) and 1 clinical study (Simon et al., 2019). Information from the U.S. veteran studies is presented separately in all of the results below because women represent a very small proportion of the overall U.S. veteran population.

The non-veteran study samples ranged in size from 58 to 7935 (*M* = 709.73, *SD* = 1247.21) and the proportions of women ranged from 24.51% to 70.74% (*M* = 47.73, *SD* = 13.50). Across the veteran studies, sample sizes ranged from 178 to 784 (*M* = 514, *SD* = 251.08) and the proportion of women ranged from 11.24% to 17.34% (*M* = 14.76, *SD* = 2.82).

### 3.2 Community vs. clinical recruitment

The average proportion of community-recruited women across the non-veteran studies was 51.20% (*SD* = 11.78%; *k* = 31) and the average proportion of clinically-recruited women across studies was 35.81% (*SD* = 12.70; *k* = 9), a difference that was statistically significant (*p* = 0.001). Across the community-recruited veteran studies, the average proportion of women was 15.90% (*SD* = 1.91; *k* = 3) while women comprised 11.24% of the single clinically recruited veteran study (no test was run due to the small number of studies).

### 3.3 Proportions of women in the trials benchmarked with country-specific AUD data

**Fig. 2:**
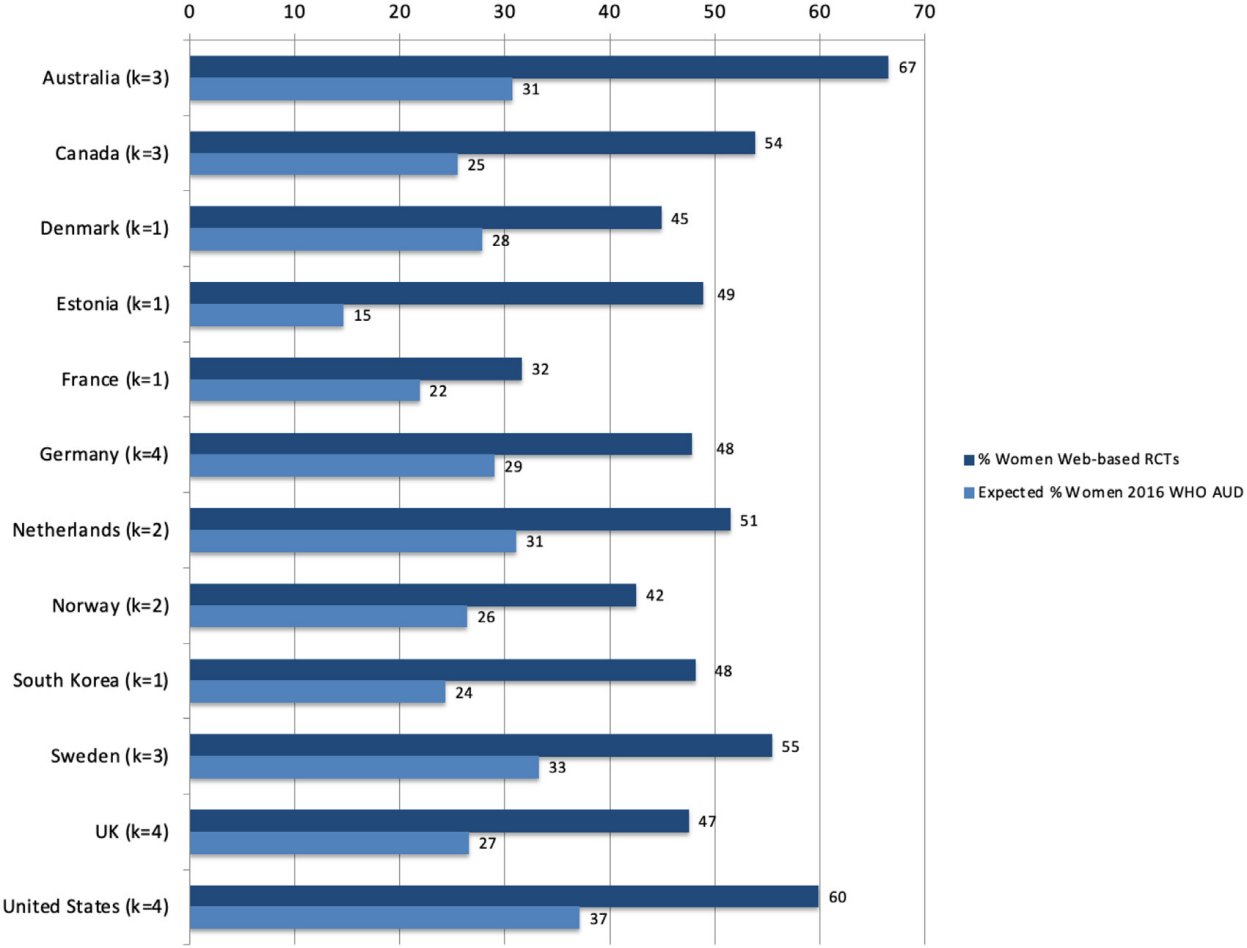
The average proportions of women in study samples compared to the respective country-specific expected proportions women with an AUD (World Population Review, 2022).

The average proportion of women in the non-veteran community studies was calculated by country and was as follows: Australia 66.52% (*k* = 3); Canada 53.77% (*k* = 3); Denmark 44.86% (*k* = 1); Estonia 48.90% (*k* = 1); France 31.56% (*k* = 1); Germany 47.80% (*k* = 4); Netherlands 51.44% (*k* = 2); Norway 42.48% (*k* = 2); South Korea 48.13% (*k* = 1); Sweden 55.39% (*k* = 3); United Kingdom 47.47% (*k* = 4); and U.S. 59.87% (*k* = 4). As may be seen in Fig. 2, when the average proportions of women in the study samples are benchmarked against their respective country-specific expected proportions of those with an AUD who are women (World Population Review, 2022), the discrepancies range from 10% (France) to 36% (Australia). These discrepancies were all in the direction of greater than expected proportions of women included in the online intervention trials. Overall, across the represented countries, the expected proportion of those with an AUD who are women (World Population Review, 2022) is 27% while across the community-based online AUD intervention trials 51.2% of the participants were women.

As noted in the Methods section, the benchmarking for the three studies of community-recruited U.S. veterans necessarily included the extra step of accounting for the proportion of veterans who are women such that among U.S. veterans with an AUD, approximately 3.49% would be expected to be women. Across the three community-recruited online alcohol RCTs focused on U.S. veterans (who all met the hazardous drinking criteria), the average proportion of women was 15.90%.

### 3.4 Gender-focused recruitment efforts and gender-tailored alcohol inclusion criteria

Only two of the 44 studies indicated having specifically recruited women. Jo and colleagues (2019) ensured that the proportions of women and men in the sample were similar to the proportions of women and men in the general South Korean population. Simon and colleagues (2019) recruited veterans from both VA general primary care clinics and women's primary care clinics. Given the small number of such studies, we were unable to statistically compare the proportions of women in studies that did and did not recruit specifically for women and simply note that 42 of the 44 studies identified did not use recruitment strategies focused on increasing women's representation in the trials.

Twenty-two of the included studies used gender-tailored alcohol inclusion criteria (e.g., AUDIT-C ≥ 4 for women, AUDIT-C ≥ 5 for men). Of the remaining 22 studies, two used DSM-IV-R or DSM-5 AUD criteria, one queried participants’ concerns about their alcohol use, and 19 used non-tailored drinking or AUDIT-C criteria, all but one of which (Cunningham et al., 2009) were consistent with unsafe drinking thresholds for men, not women. Excluding the veteran studies and those that used AUD diagnostic criteria or self-concern about drinking, we compared the studies that did (*k* = 18) or did not (*k* = 19) use gender-tailored alcohol entry criteria. The proportion of women did not differ significantly based on the use of gendered inclusion criteria, *F*(1, 35) =0.056, *p* = 0.81; the mean percent of women in studies that used vs. did not use gender-tailored criteria was 47.08% and 48.11%, respectively.

### 3.5 Changes over time in the proportion of women

Among the community-recruited samples, 22 provided information on the year or years during which recruitment took place with the most recent having completed recruitment at the end of March 2020 (Baumgartner et al., 2021). We did not discern a temporal pattern in the proportion of women included in these studies over time but these results were limited by missing data and study coverage gaps between 2005 and 2020.

### 3.6 Participant characteristics and outcomes by gender

Only one study (Hansen et al., 2012) reported participant characteristics at baseline by gender. In this study, men reported drinking, on average, 32 drinks/week to women's 21 drinks/week and they were also more likely to report weekly (gender-calibrated) binge drinking (men: 49.9%; women: 25.5%). Men were somewhat older than women, were more likely to be married and were more likely to be smokers. Men and women did not differ appreciably on education level or employment status. All other studies provide baseline characteristics for their entire samples, often stratified by intervention condition at baseline, but not by gender.

Five studies reported alcohol outcomes by gender (Hansen et al., 2012; Khadjesari et al., 2014; Miller et al., 2018; Pedersen et al., 2017; Tait et al., 2019; Wallace et al., 2011), representing 11% of the identified studies. Hansen and colleagues (2012) reported decreases in drinks per week by both men and women from baseline to six-month follow-up regardless of intervention condition. Although formal tests of differences in outcomes between genders were not reported, consumption decreased, on average, approximately 23% for women and 19% for men. Miller and colleagues (2018) reported decreases in average drinks per week, binge frequency, and alcohol-related consequences across all conditions for both men and women from baseline to one-month follow up. While interactions between condition and gender were not reported, the most severe women (those who reported blackouts at baseline) assigned to the experimental condition improved proportionally more than their male peers; 66% and 51% reduction in binge drinking by women and men, respectively. In the control condition, women and men who experienced blackouts decreased their binge drinking by 28% and 35%, respectively.

The other three studies (Pedersen et al., 2017; Tait et al., 2019; Wallace et al., 2011) evaluated whether gender interacted with condition (and time) to influence outcomes. Tait and colleagues (2019) reported that assignment to the experimental vs. the control condition did not influence outcomes and that there was no condition by time by sex interaction. Wallace and colleagues (2011) also found no significant between-group differences in comparisons of their experimental and control conditions and a condition-by-gender interaction yielded no statistically significant differences in alcohol consumption between men and women during any follow up assessment. Pederson and colleagues (2017) also tested the moderating effect of gender on intervention group and reported no statistically significant differences in alcohol outcomes. Thus, across all three of these studies, men and women were found to have responded to intervention and control conditions in similar fashions.

## 4 Discussion

In the present systematic review, we evaluated possible drivers of the apparent disproportionate enrollment of women in online alcohol interventions first noted in Riper et al. (2018) person-level meta-analysis of such interventions. We identified 44 studies that recruited both women and men into RCTs evaluating online alcohol interventions of various types, intensity, and duration. Omitting the four studies that recruited only U.S. veterans from analysis, the proportion of women ranged from 24.72% in a clinical sample of German medical inpatients (Freyer-Adams et al., 2018) to 70.74% in an Australian community-based sample (Tait et al., 2019). In order to gage the extent to which women are over-represented in community-based trials, we used WHO rates of AUD (World Population Review, 2022) to estimate the expected proportion of women who “should” be enrolling in online alcohol interventions and compared these country-specific prevalence rates to the average percentage within each country of women that enrolled in the community-based RCTs.^6^ We found that women were over-represented in online alcohol RCTs nearly two-fold relative to what would be expected based on the average WHO AUD proportions of those with AUD who are women across the countries represented in the trials (online alcohol RCTs: 51.20%; WHO: 27.0%). In our separate review of veteran studies, we found that the average proportion of U.S. women veterans in these RCTs (15.90%) is over four-fold greater than would be expected based on the proportion of U.S. veterans with AUD who are women (3.49%), which may be a reflection of some women veterans’ challenges obtaining alcohol treatment in male-dominated VA healthcare settings (Giannitrapani et al., 2018) or the dearth of women-only VA addiction programing (Timko et al., 2017). Thus, it appears safe to conclude that both non-veteran and veteran women are disproportionately participating in online alcohol intervention trials.

At the outset of this investigation, we knew that if the over-representation of women in these trials found by Riper et al. (2018) persisted in the larger universe of relevant studies, it was critical to ascertain whether their over-representation appeared to be driven by mundane trial design factors rather than simply concluding that women are differentially drawn to online alcohol interventions. Thus, we evaluated whether studies that reported making specific efforts to recruit women or that used gender-tailored alcohol inclusion criteria (or a combination) had significantly greater proportions of women than studies that did not have these trial design factors. We somewhat surprisingly found that only 2 of the 44 studies reported targeting recruitment by gender so this was almost certainly not a factor in the over-representation of women in these trials. Similarly, the proportions of women in trials that did and did not use gender-tailored alcohol inclusion criteria were not significantly different, and indeed, were nearly identical. This pattern of findings strongly suggests that women are not over-represented in these trials as an artifact of studies using gender-tailored norms, which have lower drinking thresholds for women. Rather, consistent with Riper et al. (2018) finding that women were, on average, likely to have more serious alcohol problems than men at baseline, the present findings suggest that not only are women engaging in online alcohol interventions at rates well-beyond what would be expected from population base rates, but they are turning to these interventions when their drinking levels and/or related consequences are quite serious. Thus, neither of these study design factors explain, or even help to explain, women's marked over-representation in online alcohol intervention trials. Additionally, in light of Magill et al. (2019) finding that between 1980 and 2018, women comprised only 30% of the participants enrolled across 30 randomized controlled trials of in-person cognitive-behavioral interventions for alcohol and drug use disorders, it appears unlikely that the current findings are an artifact of women differentially volunteering for research trials.

Of note, only one study's recruitment period overlapped at all with the onset of the COVID-19 pandemic, having recruited from February 2016 through March 2020 (Baumgartner et al., 2021). Thus, the disproportionate inclusion of women in online alcohol trials is also not explained by either COVID-related increases in excess or unsafe drinking among women (Barbosa et al., 2021; Pollard et al., 2020; Rodriguez et al., 2020) or lack of access to traditional alcohol treatment (Gibson et al., 2022; Jackson et al., 2021; Livingston et al., 2022; Narasimha et al., 2022), both of which are important public health issues whose impact on the demand by women for online alcohol treatment options is as yet unknown.

Unfortunately, our systematic review of this literature yielded no tangible clues as to what is driving women's over-representation in online alcohol intervention trials. To date, there does not appear to be an effort to find out from people who participate in online alcohol interventions why they chose them, if they chose them over traditional, therapist-led interventions, and whether there are noteworthy differences between women and men on these factors. The answers to these questions could help the field better target and tailor such interventions to the needs of women, who as demonstrated, are disproportionately likely to seek them out and may be even more likely to seek them out in the years to come given the aforementioned COVID-19-related issues. Specifically, it would be useful to know whether women are more likely than men to cite secrecy about alcohol concerns, stigma, lack of support from their social networks to change their drinking behaviors, challenges attending traditional treatment due to childcare responsibilities and/or work schedules, and/or lack of funding or insurance to pay for care (Grella, 2009; Haller et al., 2003; Lale et al., 2014; McCrady et al., 2020; McHugh et al., 2018; Substance Abuse and Mental Health Services Administration, 2009; C. Tuchman, 2010; Verissimo et al., 2017) as reasons for participating in online treatment options. Many of these issues were especially exacerbated for women during the COVID-19 pandemic, suggesting that in the years to come, women will likely contend with elevated mental health and substance use challenges stemming from job loss, financial strain, childcare challenges, gender-based violence, and worsening mental health (Badri, 2020; Burki, 2020; Ewing-Nelson, 2020; The Lancet Editorial Board, 2020; Peck, 2020; 2021), and that treatment systems will need to use innovative methods, such as online platforms, to meet these needs (Murthy and Narasimha, 2021).

Importantly, while the field is indebted to Riper et al. (2018) for the person-level meta-analysis that allowed us to see that overall women do not appear to be benefiting as much as men from online alcohol interventions, overall only 11% of the available studies and only one study published nearly contemporaneously (Tait et al., 2019) have evaluated whether gender interacts with treatment condition to influence outcomes while none of the eight studies published since 2020 have done so (Augsburger et al., 2022; Baumgartner et al., 2021; Mujcic et al., 2020; Sanatkar et al., 2021; Schaub et al., 2021; Stapinski et al., 2021; Sundström et al., 2020a, 2020b). Thus, it would be very helpful if future investigations of online alcohol interventions tested gender x time x condition interactions *and* reported gender-specific baseline and follow-up outcome information as would such secondary analyses of extant trials. It would be especially useful for the field to examine whether more vs. less intense online interventions (number of modules, length of engagement) are differentially effective for women and/or men. For instance, gender differences are not typically observed in standard, in-person behavioral AUD interventions, which are more intensive than online treatments and involve multisession coping skills and/or relapse prevention training (see Magill et al., 2019 Supplemental Material #2). Given the wide range of intensity and duration of online interventions, with many involving single-session assessment and normative feedback only, future research is needed to evaluate whether the observed gender differences relate to the modality of the treatment (in person vs. online), duration or intensity of the intervention, or both. Likewise, we found that only one of the 44 studies reported baseline participant characteristics by gender (Hansen et al., 2012). Thus, we also encourage future studies to provide this information so that the field can better appreciate the differences and similarities of women and men who choose online alcohol interventions to address their drinking concerns.

The strengths and weaknesses of the current systematic review are largely, though not entirely, related to the strengths and weaknesses inherent to the literature from which it draws. Regarding strengths, first, we were able to identify more than three times as many RCTs that evaluated online alcohol interventions with both women and men than Riper et al. (2018) included, largely because a great many were published after the earlier study's search timeframe concluded and the earlier study only included studies whose authors provided access to their trial data. Second, the greatly expanded number of relevant trials not only allows us a measure of confidence in the findings, but it also enabled a more nuanced look at the types of samples that were recruited. Specifically, we can now see that when people are recruited for these trials from clinical settings, women are somewhat over-represented relative to their expected proportion (clinical studies proportion that is women: 35.81%; WHO AUD proportion that is women: 27%) while when they are drawn from the community, they are markedly over-represented (community studies proportion that is women: 51.20%). Third, this study was strengthened by the use of country-specific WHO AUD rates in that they provided a way to create expected proportions of women with alcohol concerns to compare with the proportions in the online alcohol RCTs. Though imperfect in that most of the extant online alcohol intervention trials did not require an AUD diagnosis for study entry, without such a benchmark it would be impossible to gage just how remarkable the proportions of women in these trials is. Of note, two community-recruited trials (Cougle et al., 2017; Sundstrom et al., 2020a) did require participants to meet DSM-5 AUD criteria and the proportions of women were 68.97% (Cougle) and 51.20% (Sundstrom).

In addition to the lack of pre-registration, a central weakness of this systematic review is that we were limited to the information available in the published reports of the trials and did not have access to the person-level data. Although we were able to address our primary methodological questions regarding gender-targeted recruitment and gender-tailored alcohol inclusion criteria, we were not able to address the important question of whether women are not benefiting as much from online alcohol interventions as men in this larger set of studies given that only 5 of the 44 identified studies reported gender-relevant outcome information. We are also unable to speak to whether the women who came into this larger set of trials continue to present with somewhat more severe alcohol involvement than the men as Riper et al. (2018) or why women (and men) seek out online treatment trials.

## 5 Conclusion

In this systematic review we evaluated articles featuring results of online alcohol interventions that included women and men. Our objective was to evaluate the proportion of women included in these studies and trial design features to gain a better understanding of whether trial design features (i.e., gender-specific recruitment and/or alcohol inclusion criteria) or women's self-selection into trials was more likely driving their disproportionate involvement. We found that women are indeed significantly overrepresented in online alcohol interventions with US women veterans being especially overrepresented, and that this phenomenon seems to be driven more by women's self-selection than by trial design features, suggesting greater interest or perhaps need on the part of women than men for self-help tools that circumvent common barriers to care (e.g., stigma, childcare, financial constraints, lack of health insurance). In light of the current findings and Riper et al. (2018) person-level meta-analytic finding that online alcohol interventions are less efficacious for women than for men, future research in this area should undertake the development and rigorous evaluation of gender-responsive online alcohol interventions to better meet women's needs and that result in improvements for them that are at least on par with men's. Moreover, we urge stakeholders (e.g., governmental, philanthropic entities, etc.) to make such interventions available outside of traditional fee for service treatment venues (i.e., at no cost) to decrease barriers associated with finances, insurance coverage, and stigma (Coombs et al., 2021; Martin et al., 2022) and to circumvent the lack of available health care providers (Murthy and Narasimha, 2021; Narasimha et al., 2022).

## Declaration of Competing of Interest

No conflict declared.

## Footnotes

1 Overall, across the 19 studies included in the meta-analyses women comprised 47.6% of the participants but three of the studies recruited only men and when those studies are not considered, the proportion of women is 52%.

2 One study included by Riper et al. (2018) evaluated a telephone-based intervention (Bischof et al., 2008) and one evaluated a text messaging intervention (Suffoletto et al., 2014) and these studies were not included in the current review.

3 Secondary analysis papers that provided information on participant characteristics or outcomes by gender would have been used in conjunction with the original outcome paper, but no such papers were found.

4 We also examined 2016 WHO country-specific data on harmful alcohol use as a potential benchmark and while there were some country-specific discrepancies between this and the rates of AUD, overall they were in very good agreement so to be more parsimonious we are presenting only the AUD information.

5 Information for 2016 is not available.

6 For this benchmarking exercise, we excluded studies that recruited from multiple countries as well as those that recruited solely from clinical settings so as not to conflate those who had already sought treatment with uptake of online alcohol treatment.
